# Lactobacillus Modulates Chlamydia Infectivity and Genital Tract Pathology *in vitro* and *in vivo*

**DOI:** 10.3389/fmicb.2022.877223

**Published:** 2022-04-28

**Authors:** Hongliang Chen, Shuling Min, Li Wang, Lanhua Zhao, Fangzhen Luo, Wenbo Lei, Yating Wen, Lipei Luo, Qianting Zhou, Lixiu Peng, Zhongyu Li

**Affiliations:** ^1^Chenzhou No.1 People’s Hospital, Hengyang Medical School, University of South China, Chenzhou, China; ^2^Institute of Pathogenic Biology, Hengyang Medical College, Hunan Provincial Key Laboratory for Special Pathogens Prevention and Control, Hunan Province Cooperative Innovation Center for Molecular Target New Drug Study, University of South China, Hengyang, China; ^3^Chenzhou No.1 People’s Hospital, The First School of Clinical Medicine, Southern Medical University, Chenzhou, China

**Keywords:** chlamydia, lactobacillus, D (–) lactic acid, infectivity, pathology

## Abstract

Since we previously reported that women infected with chlamydia had a significant overall reduction in Lactobacillus in the vagina microbiota as compared to those uninfected individuals; the interactions between the altered Lactobacillus and *Chlamydia trachomatis*, on the other hand, need to be elucidated. Here, we employed both *in vitro* and *in vivo* models to evaluate the effects of this changed Lactobacillus on Chlamydia infection. We found that *L. iners, L. crispatus, L. jensenii, L. salivarius, L. gasseri, L. mucosae*, and *L. reuteri* all significantly reduced *C. trachomatis* infection in a dose- and time-dependent manner. The strongest anti-Chlamydia effects were found in *L. crispatus* (90 percent reduction), whereas the poorest was found in *L. iners* (50 percent reduction). D (–) lactic acid was the key component in Lactobacillus cell-free supernatants (CFS) to inactivate Chlamydia EBs, showing a positive correlation with the anti-Chlamydia activity. The effects of D (–) lactic acid were substantially attenuated by neutralizing the pH value to 7.0. *In vivo*, mice intravaginally inoculated with Lactobacillus mixtures (*L. crispatus*, *L. reuteri*, and *L. iners* at a ratio of 1:1:1), but not single Lactobacillus, after genital Chlamydia infection, significantly attenuated the levels of Chlamydia live organism shedding in both the lower genital tract and the intestinal tract, reduced cytokines production (TNF-α, IFN-γ, and IL-1β) in the vagina, and lessened upper genital tract inflammation and pathogenicity. Taken together, these data demonstrate that Lactobacillus inhibits Chlamydia infectivity both *in vivo* and *in vitro*, providing useful information for the development of Lactobacillus as adjunctive treatment in Chlamydia infection.

## Introduction

*Chlamydia trachomatis* is a Gram-negative, obligate intracellular bacterium, sharing a unique biphasic developmental cycle alternating between the intracellular, non-infectious reticulate body (RB) and the extracellular, infectious elementary body (EB) (Bastidas et al., 2013; Elwell et al., 2016). Clinical isolation of *C. trachomatis* from the eyes of patients was first achieved by a Chinese scientist named Feifan Tang (Tang et al., 1957), speeding up chlamydia research. *C. trachomatis* can cause trachoma, which is the leading infectious cause of non-congenital blindness (Lansingh, 2016). Thereafter, the role of *C. trachomatis* in genital tract disease attracted extensive attention worldwide. The genital tract biovar (serovars D–K) was recognized as the most prevalent identified cause of sexually transmitted diseases (Rawre et al., 2017), with estimates of over 131 million new cases occurring annually worldwide (Kreisel et al., 2021). Most chlamydial infections of the female genital tract are asymptomatic and often get unnoticed. Indeed, depending on the nature of the immune response, some individuals can clear up their infection, whereas 15–40% of *C. trachomatis* infection ascends to the upper genital tract and spreads to the uterus and fallopian tubes, leading to serious health consequences, including pelvic inflammatory disease, ectopic pregnancy, and infertility (Golden et al., 2005; Geisler, 2010).

*Chlamydia trachomatis* has been described as a symbiont with unique cell biological features; they seemed not to parasitize any cells in the vaginal wall, which partially explained that *C. trachomatis* infection usually did not cause vaginitis (Winkler and Crum, 1987). Besides, the vagina is home to a plethora of microorganisms, establishing the vaginal micro-ecosystem and maintaining a functional equilibrium. A healthy equilibrium formed a mutually beneficial relationship with their hosts and provided a barrier to pathogenic organisms (Chee et al., 2020). Pathogenic bacterial infections, including *Neisseria gonorrhoeae*, HPV, and *mycobacterium tuberculosis* infection, can cause a dysbiosis of the vaginal microbiome (Kayukova et al., 2019; Tamarelle et al., 2019), which, in turn, is thought to influence the outcome of their infections.

As is the same with *C. trachomatis*, our previous work has revealed that women with *C. trachomatis* infection had an *L. iners*- rather than *L. crispatus*-dominated vaginal microbiota and presented a remarkable decrease in Lactobacillus (Chen et al., 2021). A few studies have reported the inhibitory effects of Lactobacillus on *C. trachomatis*, the possible mechanisms involved which might include interference with expressions of α5 integrin to affect adhesion or the entry process, production of lactic acid or other metabolites able to inhibit *C. trachomatis*, and reduction of the cytokines production (Mastromarino et al., 2014; Rizzo et al., 2015; Sessa et al., 2017; Parolin et al., 2018). We thus conducted this study to further investigate the role of the top seven altered Lactobacillus strains on the outcome of *C. trachomatis* infection both *in vitro* and *in vivo*. In particular, we have examined the anti-chlamydial activity of *L. iners, L. crispatus, L. jensenii, L. salivarius, L. gasseri, L. mucosae*, and *L. reuteri in vitro*, analyzed the anti-microbial product of lactobacillus, and evaluated the effects of single or mixed lactobacillus on *Chlamydia-*mediated pathogenicity *in vivo*. Our data have demonstrated the following: (i) *L. crispatus* has an excellent ability to inactivate the infectivity of *C. trachomatis* in a dose and time-dependent manner; (ii) D (–) lactic acid is the principal component in lactobacillus culture supernatants to inactive *C. trachomatis via* an acid-dependent mechanism; and (iii) mixed lactobacillus intravaginal administration attenuates chlamydia-mediated pathogenicity in the upper genital tract, indicating a new probiotic strategy to treat sexually transmitted *C. trachomatis* infection.

## Materials and Methods

### Bacterial Strains and Growth Conditions

Bacterial strains were propagated as previously described (Yoshimura et al., 2020). *L. iners* (ATCC 55195), *L. salivarius* (ATCC 11741), *L. reuteri* (ATCC 23272), *L. gasseri* (ATCC 33323), and *L. mucosae* (GDMCC 801225) were purchased from Guangdong Microbial Culture Collection Center, while *L. crispatus* (ACCC 03956) and *L. jensenii* (ACCC 03962) from China General Microbiological Culture Collection Center. Strains of lactobacillus were grown in an MRS medium with minor modifications at 37°C in a humidified 5% CO_2_ incubator under anaerobic conditions. Recovered Lactobacilli were discarded after 15 passages, following the guidance.

*Chlamydia trachomatis* serovars E and *C. muridarum* Nigg strain organisms were propagated in HeLa cells (ATCC CCL-2.1) and cultured in DMEM (Dulbecco’s Modified Eagle Medium; Gibco, NY, United States), supplemented with 10% (v/v) fetal bovine serum (FBS; Gibco, NY, United States) at 37°C in a 5% CO_2_ incubator. Chlamydia EBs were harvested, purified, and titrated as described previously (Huo et al., 2020). Aliquots of live EB organisms were stored at −80°C in a sucrose-phosphate-glutamic acid (SPG) buffer, consisting of 218-mM sucrose, 3.76-mM KH_2_PO4, 7.1-mM K_2_HPO4, and 4.9-mM glutamate (pH 7.2) till use.

### Determination of Lactobacillus Metabolites and Lactic Acid Concentrations in Culture Supernatants

Lactobacilli were cultured for 24–36 h until an OD600 of 2.0 (5- × −10^8^ colony forming unit/ml) was achieved. Lactobacilli cultures were centrifuged at 3,000 × *g* for 10 min at 4°C, and the supernatants were collected and sterilized by passing through 0.2-μm membrane filters. Lactobacilli cell-free supernatants (CFS) were then obtained and stored at −20°C till use (4°C for immediate use). pH values of lactobacillus CFS were measured using a Fisher pH meter, while 45 lactobacillus metabolic components, including lactic acid, non-derivatization of various amino acids, and carnitine concentrations of which were assessed using ion-selective electrode and ultra-performance liquid chromatography tandem mass-spectrometry (UPLC-MS/MS) (Waters, MA, United States) according to the manufacturer’s instructions. Lactic acid isomers [D (–) and L (+) lactic acid] concentrations of lactobacillus CFS were determined using the lactate assay kit (Eton Bio, CA, United States) based on the reduction of the tetrazolium salt INT in a NADH-coupled enzyme reaction to formazan.

### *Chlamydia trachomatis* Infectivity Assays

Ten microliters of *C. trachomatis* EBs with a multiplicity of infection (MOI) of 1 were exposed to 90 μL of either various lactobacillus CFS, including three different CFS concentrations (1:1, 1:10, and 1:100 dilutions) and pH-adjusted CFS, 10-mM lactic acid isomers, hydrochloric acid, a DMEM medium, or an MRS medium in a 1.5 ml tube for 7 min to 1 h at 37°C. The mixtures were used to infect 2- × −10^6^ HeLa cells by centrifugation at 600 × *g* for 1 h in a 12-well plate, containing a 2-ml DMEM medium to facilitate cell penetration. The cell monolayers were then cultured in the DMEM medium supplemented with 10% FBS and cycloheximide 1 μg/ml for 36 h at 37°C in 5% CO_2_. In some experiments, *C. trachomatis* EBs were pre-incubated with *L. iners* CFS, adding L (+) or D (–) lactic acid up to 10 mM to identify a possible synergic effect with lactic acid. Chlamydia IFUs were counted by a fluorescence microscope ECLIPSE TE2000-5 (Nikon, Tokyo, Japan) using a monoclonal antibody against the *C. trachomatis* major outer membrane protein antigen conjugated with fluorescein (Invitrogen, CA, United States), as described previously (Li et al., 2008). Five random fields were viewed per coverslip. The entire coverslips should be viewed if the coverslips were with less than 1 IFU/field. In addition, the corresponding lactobacillus CFS, lactic acid isomers, and hydrochloric acid were added to HeLa cells and incubated for 36 h to evaluate their cytotoxic effect on the HeLa cells. Following incubation, the cell viability was assayed by the MTT method according to the manufacturer’s instructions.

### Experimental Mouse Model

Female BALB/c mice were purchased at the age of 5–6 weeks and weighed between 15 and 25 g from Hunan SJA Laboratory Animal Co. Ltd (License No. SCXK 2016-0002, China). The protocol used in this study was approved by Animal Use and Ethics Committee of the University of South China (Hengyang, China). Thirty mice were randomly divided into six groups of five animals in each group. All the mice were allowed to acclimatize for 4 days, followed by subcutaneous injection with 2.5-mg medroxyprogesterone (Xianju Pharma Co. Ltd, Zhejiang, China). Five days later, each mouse was intravaginal inoculated with SPG (control) alone, or 2- × −10^5^ inclusion-forming units (IFU) of live *C. muridarum* in 15-μL SPG. On Day 3 postinfection, the mice were given either an MRS medium (Cm), 15 μL of 2- × −10^9^ CFU of *L. crispatus* (Cm + LC), *L. reuteri* (Cm + LR), *L. iners* (Cm + LN) or a mixture of these bacteria (Cm + Mix) at a ratio of 1:1:1 intravaginal inoculation. After the inoculation, vaginal and rectal swab samples were collected for monitoring live organism shedding, serum samples for detection of specific antibody production, spleen for analysis of T cell-mediated cytokine responses, and intestinal and upper genital tract tissues for evaluation of the pathology (Lu et al., 2012; Huo et al., 2020), respectively.

### RT-qPCR

Bacterium-specific RT-qPCR assays were conducted to verify lactobacillus colonization in the mouse vagina by amplifying species-specific regions of the 16S rRNA gene as described previously (Chen et al., 2021). Briefly, primers specific to lactobacillus characterized by the 16S rRNA amplicon were designed as follows: Universal primer (926 F: AAACTCAAAKGAATTGACGG, 1062 R: CTCACRRCACG AGCTGAC), Liners (Liners F: CTCTGCCTTGAAGATCG GAGTGC, Liners R: ACAGTTGATAGGCATCATCTG), *L. crispatus* (Lcris F: AGCGAGCGGAACTAACAGATTTAC, Lcris R: AGCTGATCATGCGATCTGCTT), *L. reuteri* (Lreu F: ACCGAGAACACCGCGTTATTT, Lreu R: CATAACTTAACCT AAACAATCAAAGATTGTC), and β-globin (B-GH20 F: GAA GAGCCAAGGACAGGTAC, B-PC04 R: CAACTTCATCCAC GTTCACC). Mice vaginal swabs were collected on Day 7 after chlamydia infection. Serial dilutions of 1, 1:4, 1:16, 1:64, and 1:256 vaginal swab bacterial total DNA were used to determine primer amplification efficiencies. Quantitative analysis of vaginal bacterial communities was performed using the following formula:


$$X=\frac{{\left(Eff.Univ\right)}^{C\,t\,u\,n\,i\,v}}{{\left(Eff.Spec\right)}^{C\,t\,s\,p\,e\,c}}\times 100$$

where “Ct spec” and “Ct univ” represent the cycle threshold, “Eff. Spec” represents the efficiency of various genera-specific primers, and “Eff. Univ” represents the efficiency of bacterial universal primers. “X” represents the percentage of bacterial species-specific gene copy number existing in a sample.

### Titrating Live Chlamydial Organisms Recovered From Swabs

Both vaginal and rectal swabs were taken at different time points postinoculation (one time every 3–7 days) to monitor live chlamydial organism shedding. As previously reported by Chen et al. (2010) and Huo et al. (2020), each swab was placed in 500-μL of ice-cold SPG and vortexed with glass beads to accelerate the release of chlamydial organisms. Following sonication on ice, 200-μL supernatants were inoculated onto HeLa cell monolayers in duplicate and the presence of 2-mg/ml cycloheximide. The cultures were imaged for an immunofluorescence assay under a fluorescence microscope (Huo et al., 2020). The calculated total number live organisms IFUs recovered from each swab was converted into log10 for calculating group mean and standard deviation.

### Genital and Intestinal Tract Pathologies

The gross pathology of the hydrosalpinx, mouse genital and intestinal tract tissue pathologies, and histological scoring were assayed, as has been described previously (Lu et al., 2012). Briefly, all the mice were anesthetized with ketamine (100 mg/kg) and xylazine (4 mg/kg) and sacrificed on Day 80 after intravaginal chlamydia infection. Before the tissue removal, an *in situ* gross examination was performed by an experienced technician blinded to mouse treatment for evidence of hydrosalpinx; the severity of which was further scored based on an ordinal scale. After imaging, the spleen was harvested for measuring T cell-mediated cytokine responses (Li et al., 2019). Briefly, spleen was harvested from each mouse at Day 80, and 2- × −10^6^ splenocytes were cultured with cell 1 μL of a stimulation cocktail (phorbol 12-myristate 13-acetate and ionomycin mixtures) and 0.5-μL brefeldin A (BFA) (eBioscience, Shanghai, China) at 37°C for 6 h. Following the stimulation, the cells were incubated with an Fc receptor blocking antibody (CD16/CD32 mAb) (eBioscience, Shanghai, China). After that, the cells were stained with surface markers-CD8a- PerCP-Cy5.5 and -CD4-FITC for 30 min at 4°C. Then, the cells were permeabilized with a Cytofix/Cytoperm*™* kit (eBioscience, Shanghai, China) and stained intracellularly for 30 min at 4°C for cytokines IL-4-PE and IFN-γ-APC (eBioscience, Shanghai, China). After washing, sample acquisition was performed with a BD FACS Canto II flow cytometer.

While the other excised tissues, including the oviduct, uterine horn, small and large intestines, were fixed in 10% neutral formalin, embedded in paraffin, and serially sectioned longitudinally (with 5 μm/each section), the sections were stained with H&E and scored for severity of inflammation and luminal dilation by a certified pathologist blinded to mouse treatment. Both oviduct and uterine horn were scored for inflammatory infiltration and luminal dilation, whereas small and large intestines only for inflammatory infiltration. Scores assigned to individual mice were calculated as means and standard deviations per group for statistical analyses.

### Enzyme-Linked Immunosorbent Assay

The cytokines TNF-α, IFN-γ, IL-1β, IL-6, and IL-10 in the mouse vaginal swabs were assayed using commercial Enzyme-Linked Immunosorbent Assay (ELISA) kits (Biolegend, CA, United States), following the manufacturer’s instructions as described previously (Chen et al., 2021). Briefly, each mouse vaginal swab was placed in a tube with 500-μL PBS and centrifuged at 12,000 × *g*, 30 min at 4°C. The supernatants were added to 96 well ELISA microplates, pre-coated with the corresponding capture antibodies with 50 μL/well. Sample cytokines bound to the capture antibody were detected biotinylated and horseradish peroxidase (HRP)-conjugated antibodies. Final cytokine concentrations were calculated in pg/ml from a standard curve based on absorbance values in each experiment.

For titrating chlamydia-specific antibodies, including IgG, IgG1, and IgG2a in mouse serum, 96-well plates were coated with UV-inactivated *C. muridarum* EBs as antigens (Lu et al., 2012). The antibody isotype from mouse serum was detected with HRP-conjugated goat anti-mouse IgG, IgG1, and IgG2a, respectively. The cut-off value for each experiment was determined according to the formula of the form “mean ± 3 standard deviation of negative controls.” Finally, the highest dilution of a given antiserum with positive recognition of *C. muridarum* EBs was recorded as the titer of the antiserum, as previously reported (Lu et al., 2012).

### 16S rRNA Amplicon Sequencing of Vaginal and Intestinal Samples

Both vaginal and intestinal swab samples were collected from mice pre- and post- intravaginal infection with chlamydia, stored at −20°C until use. 16S rRNA amplicon sequencing of each sample was carried out by an Illumina HiSeq platform (Novogene, Beijing, China) as previously described (Chen et al., 2021). Briefly, genomic DNA was extracted by using a Genomic DNA Mini Preparation kit (Tiangen, Beijing, China) as templates to amplify the V3–V4 hypervariable regions of 16S rRNA genes. The dominant PCR products were purified for constructing sequencing libraries, ultimately sequencing on an Illumina HiSeq platform, generating 250-bp paired-end reads. The operational taxonomic units (OTUs) clustering and species classification were analyzed at a 97% identity level based on effective data. Subsequently, the relative abundance, Alpha diversity, as well as multi-sequence alignment of OTUs, were calculated using R version 4.0.0. Phylogenetic trees and PCoA analysis were performed to explore the differences between community structure among distinct samples and groups.

### Statistical Analysis

Statistical analyses were conducted using GraphPad Prism 8.0. The distribution of the continuous variables was expressed as the mean ± standard deviation. EB titers, lactobacillus metabolite concentrations, the semi-quantitative pathology score data, cytokine production, and the area under the curve of cytokines production and the number of recover chlamydial organisms (Shao et al., 2018) were compared between groups using one-way ANOVA. The incidences of hydrosalpinx between groups were analyzed using Chi-square test. Correlations between lactic acid isomer concentrations and anti-chlamydia activity were evaluated by calculating Pearson’s correlation coefficients. A *P*-value of < 0.05 was considered significant.

## Results

### Pre-exposure to Lactobacillus Culture Supernatants Reduces *Chlamydia trachomatis* Infectivity

**FIGURE 1:**
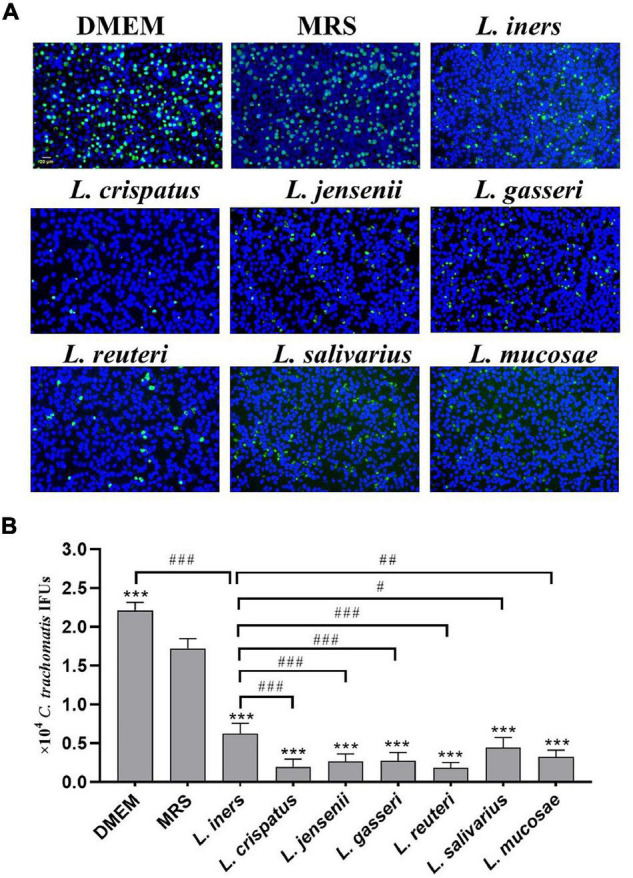
Effect of lactobacillus cell-free culture supernatants on *C. trachomatis* infectivity. *C. trachomatis* EB was pre-incubated with either a DMEM cell culture medium (control), an MRS bacterial culture medium (control), or sterile-filtered cell-free culture supernatants collected from overnight cultures of *L. iners, L. crispatus, L. jensenii, L. gasseri, L. reuteri, L. salivarius*, *and L. mucosa* prior to HeLa cells infected with *C. trachomatis*. The infected cells were processed for immunofluorescence detection of the *C. trachomatis* E organism (green), and DNA (blue) **(A)**. *C. trachomatis* IFUs were scored by fluorescence microscopy **(B)**. Each bar represents the mean ± SD of the IFUs from three independent experiments. ***p < 0.001 vs. MRS control; ^#^*p* < 0.05, ^##^*p* < 0.01, and ^###^*p* < 0.001 vs. *L. iners* supernatants treatment (one-way ANOVA).

Our previous study has demonstrated a lactobacillus species–dominated vaginal microbiome alteration among female infertility with *C. trachomatis* infection. *L. iners, L. crispatus, L. jensenii, L. salivarius, L. gasseri, L. mucosae*, and *L. reuteri* were the seven most altered lactobacilli (Chen et al., 2021), which might be associated with *C. trachomatis* infection. We, therefore, sought to deduce the potential effects of these lactobacilli on *C. trachomatis* infectivity. *C. trachomatis* EBs were pre-exposed to various lactobacillus culture supernatants collected from overnight lactobacillus cultures for 30 min. Chlamydia IFUs generated by *C. trachomatis*-infected HeLa cells were measured by immunofluorescence. As indicated in Figure 1, all the seven lactobacillus strains significantly attenuated *C. trachomatis* infection as compared to *C. trachomatis* EBs pre-exposed to an MRS medium. *L. crispatus* and *L. reuteri* culture supernatants effectively attenuated the infectivity of *C. trachomatis* EBs (about 90 and 88% reduction, respectively). In contrast, *L. iners* CFS showed a relatively weak anti-chlamydia activity (about 50% reduction). Additionally, the MRS medium significantly attenuated *C. trachomatis* infection (about 20% reduction) relative to DMEM-treated HeLa cells (Figure 1B). We thus have used the MRS medium as the control in later experiments.

Lactobacillus CFS reduced the infectivity of *C. trachomatis* in concentration- (1:1 to 1:100 dilution) and time-dependent (7–60 min) manners, which significantly inhibited the majority of *C. trachomatis* EBs after treatment with undiluted lactobacillus CFS for 60 min. Notably, the inhibitory activity was robustly decreased in the case of adjusting the lactobacillus CFS with NaOH to pH 7.0 (Supplementary Figure 1). These data suggest that proper acidity is a requisite for lactobacillus*-*mediated anti-chlamydia activity.

### D (–) Lactic Acid Is the Key Component in Lactobacillus Cell-Free Supernatants to Inactivate *Chlamydia trachomatis*

**FIGURE 2:**
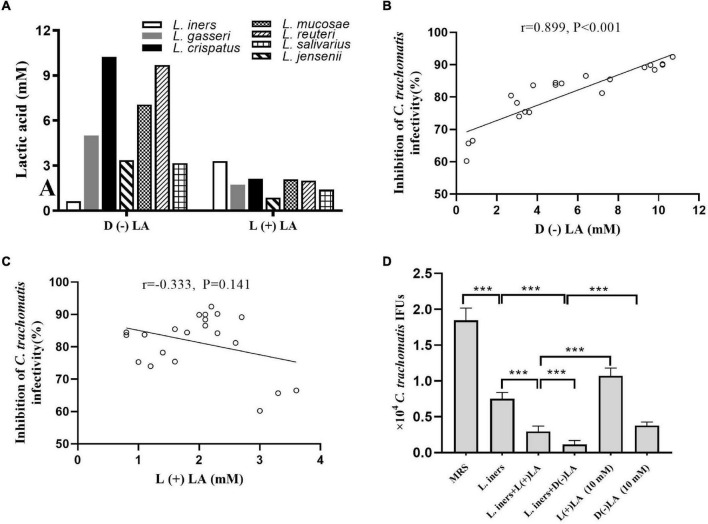
Inhibition of *C. trachomatis* by lactobacillus-produced D (−) and L (+) lactic acid. **(A)** D (−) than L (+) lactic acid concentrations in overnight lactobacillus culture supernatants. Correlation between anti-chlamydial activity and concentration of D (−) **(B)** or L (+) lactic acid **(C)**. For Pearson correlation analysis, inhibition of *C. trachomatis* infectivity (*x* axis) was plotted against the concentration of D (−) lactic acid produced by each lactobacillus (*y* axis). The correlation coefficient *r* between anti-chlamydial activity and D (−) lactic acid was 0.899, while *r* = 0.333 for L (+) lactic acid. **(D)** *C. trachomatis* EBs were pre-incubated with either an MRS bacterial culture medium (control), *L. iners* culture supernatants alone, *L. iners* culture supernatants supplemented with 9.3 mM D (−) or 6.7 mM L (+) lactic acid up to 10 mM, 10 mM D (−) or L (+) lactic acid alone. Each bar represents the mean ± SD of the IFUs from three independent experiments. Results of lactic acid isomers assays from an independent sample. ***p < 0.001 (one-way ANOVA).

Lactobacillus culture supernatants contain a wide variety of anti-microbial products. Forty-five lactobacillus metabolic components, including lactic acid, non-derivatization of various amino acids, and carnitine, were assessed using ion-selective electrodes and UPLC-MS/MS in *L. iners, L. crispatus, L. jensenii, L. salivarius, L. gasseri, L. mucosae*, and *L. reuteri* culture supernatants to gain more insight into the dominant component killing *C. trachomatis*. Lactic acid was present at 5- to 100-fold more in the seven lactobacillus culture supernatants when compared to the MRS medium, with lactic acid increasing the most among other non-derivatization of various amino acids and carnitine (Supplementary Figure 2A). We found that most lactobacillus culture supernatants had 3- to 6-fold higher D (–) than L (+) lactic acid, with the exception of *L. iners* culture supernatants, which had about 5-fold higher L (+) than D (–) lactic acid (Figure 2A). D (–) lactic acid in lactobacillus culture supernatant showed a significant positive association with the anti-chlamydia activity (*r* = 0.889, *p* < 0.001), whereas L (+) lactic acid showed no correlation (*r* = −0.333, *p* = 0.141) (Figures 2B,C), which further corroborated previous reports that D (–) lactic acid is the key mediator for this inhibitory activity (Gong et al., 2014; Nardini et al., 2016; Edwards et al., 2019).

Considering the high concentration of D (–) lactic acid in *L. crispatus* culture supernatants and its significant anti-chlamydia activity, we postulated that D (–) lactic acid was the key element in lactobacillus culture supernatants to deactivate *C. trachomatis*. To test this, we treated *C. trachomatis* EB with lactic acid isomers or pH-adjusted ones. D (–) lactic acid inhibited *C. trachomatis* infection more effectively than L (+) lactic acid; both of which inactivated *C. trachomatis* in a dose-dependent manner (2.5–15 mM) (Figure 2D and Supplementary Figure 2B). As expected, either D (–) or L (+) lactic acid can inhibit *C. trachomatis* infection at un-adjusted pH much more effectively than pH alone (HCL at pH 4), and this inhibitory activity was eliminated when the pH was neutralized to 7 (Supplementary Figure 2C), indicating that D (–) lactic acid is the active molecule for inactivating *C. trachomatis.*

To further determine the specific anti-chlamydia activity of D (–) lactic acid, we added D (−) or L (+) lactic acid with a final concentration of 10 mM to *L. iners* culture supernatants. While *L. iners* culture supernatants with supplemental D (−) lactic acid had stronger inhibitory effects than those with L (+) lactic acid, which is tantamount to *L. crispatus* culture supernatants-mediated anti-chlamydia activity. Surprisingly, stronger inhibitory properties were observed for the supplementation of *L. iners* CFS with D (−) or L (+) lactic acid than an equivalent concentration of D (−) or L (+) lactic acid alone (Figure 2D), suggesting a synergistic inhibitory activity of other molecules in the culture supernatants. In addition, both lactobacillus culture supernatants, D (−) and L (+) lactic acid did not affect HeLa cell viability (more than 90% cell viability) (Supplementary Figure 2D), ruling out any potential effect of HeLa cell viability on *C. trachomatis* infectivity. We thus demonstrated that D (−) lactic acid in lactobacillus culture supernatants contributed greatly to protecting against *C. trachomatis* infection.

### Intravaginal Inoculation With Lactobacillus Mixtures (*Lactobacillus crispatus, Lactobacillus reuteri*, and *Lactobacillus iners* at a Ratio of 1:1:1) Accelerates the Clearance of Intravaginal but Not Intestinal Chlamydial Infection

**FIGURE 3:**
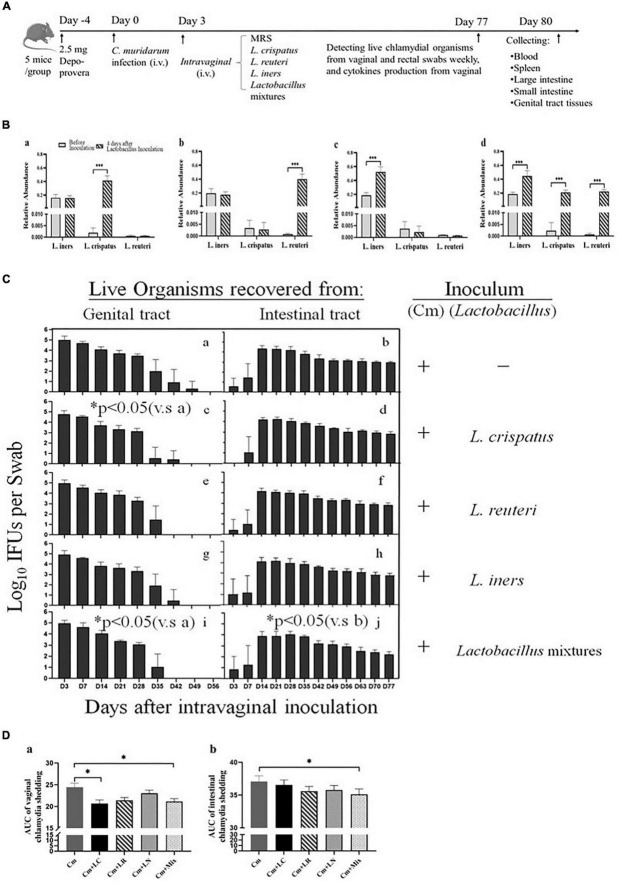
Comparison of the live organism shedding from mice intravaginal inoculated with various lactobacilli after genital chlamydia infection. **(A)** Groups of female BALB/c mice were intravaginally inoculated with 2- × −10^5^ IFUs of *C. muridarum* EBs or SPG (control) alone. As indicated on top of the figure, the mice were given either an MRS medium (Cm), 15 μL of 2- × −10^9^ CFU of *L. crispatus* (Cm + LC), *L. reuteri* (Cm + LR), *L. iners* (Cm + LN) or a mixture of these bacteria (Cm + Mix) at a ratio of 1:1:1 intravaginal inoculation 3 days after chlamydia infection. **(B)** Three days after the chlamydia infection, 15 μL of 2- × −10^9^ CFU of *L. crispatus* **(a)**, *L. reuteri* **(b)**, *L. iners* **(c)** or a mixture of these bacteria **(d)** at a ratio of 1:1:1 was intravaginally inoculated. On Day 7, vaginal swabs of each mouse were collected for detecting relative abundance of *L. crispatus, L. mucosae* and *L. iners* using qRT PCR. **(C)** Both vaginal **(a,c,e,g,i)** and rectal **(b,d,f,h,j)** swabs were taken at different time points postinoculation as indicated along the *x* axis to monitor live chlamydial organism shedding. The live organisms recovered from swabs were expressed as the log10 number of IFU per swab (*y* axis). Each group had 5 mice, and the data were obtained from 2 independent experiments. **(D)** The area under the curve of vaginal **(a)** and intestinal **(b)** chlamydia shedding. *p < 0.05, the area under the curve, compared to mice intravaginally inoculated with *C. muridarum* alone (one-way ANOVA). ***p < 0.001.

Lactobacilli have exerted robust anti-chlamydial activity *in vitro*. Subsequently, we asked whether they might play a similar role in the protection against *C. trachomatis* infection in a mouse model of genital chlamydia infections. All the mice were challenged intravaginally with *C. muridarum* organisms or SPG alone (Figure 3A). Three days after infection, the other four groups were intravaginally inoculated with *L. crispatus*, *L. reuteri*, *L. iners*, and a mixture of these bacteria at a ratio of 1:1:1, which colonized the mouse vagina, verifying through bacterium-specific RT-qPCR assays as described previously (Figure 3B). Vaginal and rectal swabs were taken every 3 or 7 days for monitoring the shedding of infectious chlamydial organisms. By Days 42–49, all the lactobacillus-administrated mice cleared vaginal infection, while the mice challenged with *C. muridarum* alone cleared vaginal infection by Day 56 postinfection. The lactobacillus mixture-administrated mice displayed a comparable number of chlamydia IFUs recovered from vaginal swabs to single lactobacillus and control up to Day 28 after infection. While, at Day 35, lactobacillus mixture and *L. crispatus* groups have reduced shedding as compared to control, *L. reuteri*, and *L. iners*. At Day 42, *L. reuteri* has less shedding than *L. iners*. Lactobacillus mixture or *L. crispatus* but not *L. reuteri or L. iners*-inoculated mice developed a significantly reduced vaginal shedding course compared to those challenged with *C. muridarum* alone, indicating lactobacillus treatment could reduce live organism shedding and increase the rate of clearance of intravaginal chlamydial infection. Chlamydial organisms could be detected in rectal swabs from a few animals (one or two in each group) as early as on Day 3 postinfection, and all the mice on Day 14, leading to robust colonization in the intestinal tract with a time course of up to 77 days, longer, consisted in a previous report (Huo et al., 2020). Single lactobacilli were comparable to the parallel group of mice intravaginally inoculated with *C. muridarum* alone, while the lactobacillus mixture-inoculated mice seemed to shed a lower level of live organisms in the intestinal tract (Figures 3C,D). These data indicate that *C. muridarum* has established robust colonization in the genital tract and spread to the intestinal tract for long-lasting colonization, and lactobacillus mixture may reduce chlamydia spreading and intestinal colonization.

### Intravaginal Inoculation With Lactobacillus Mixtures Attenuates *Chlamydia muridarum* Pathogenicity in the Upper Genital Tract

**FIGURE 4:**
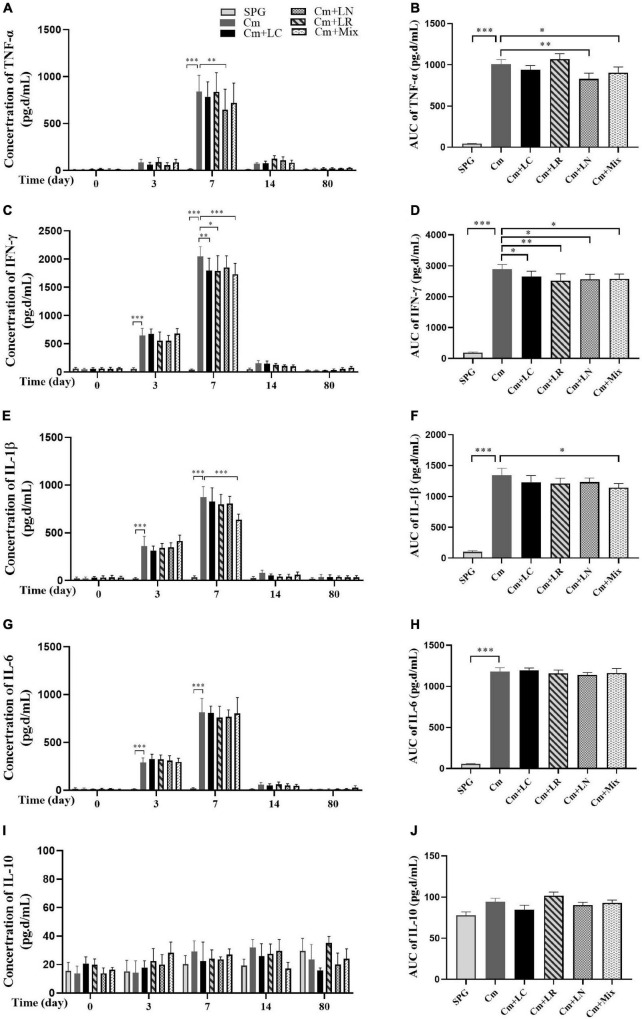
Monitoring early cytokine production kinetics from mouse genital tract after genital chlamydia infection. Vaginal swabs were taken at different time points postinoculation (Days 0, 3, 7, 14, and 80) as indicated along the *x*-axis. The levels of TNF-α **(A)**, IFN-γ **(C)**, IL-1β **(E)**, IL-6 **(G)**, and IL-10 **(I)** in the genital tract of mice were measured and expressed as pg/ml along the *y*-axis, with the corresponding area under the curve of which calculated on the right side **(B,D,F,H,J)**. Results from an independent sample. *p < 0.05, ^**^p < 0.01, and ^***^p < 0.001 vs. the Cm group (one-way ANOVA).

To investigate the effects of lactobacillus on *C. muridarum*-induced inflammatory responses *in vivo*, the production of TNF-α, IFN-γ, IL-1β, IL-6, and IL-10 in the urogenital tract of the mice at different time points after the intravaginal infection were measured. As early as Day 3, the TNF-α, IFN-γ, IL-1β, and IL-6 level significantly increased over the baseline in single or mixed lactobacilli, but not the SPG control group, and rapidly reached the peak by Day 7 postinfection (Figure 4). These cytokines production kinetics were very similar in each group, with TNF-α, IFN-γ, and IL-1β levels in the lactobacillus mixture-inoculated mice generally being lower in magnitude than those in the mice intravaginally inoculated with *C. muridarum* alone. In contrast, IL-10 production was minimal in genital tract secretions, with no differences among all groups.

**FIGURE 5:**
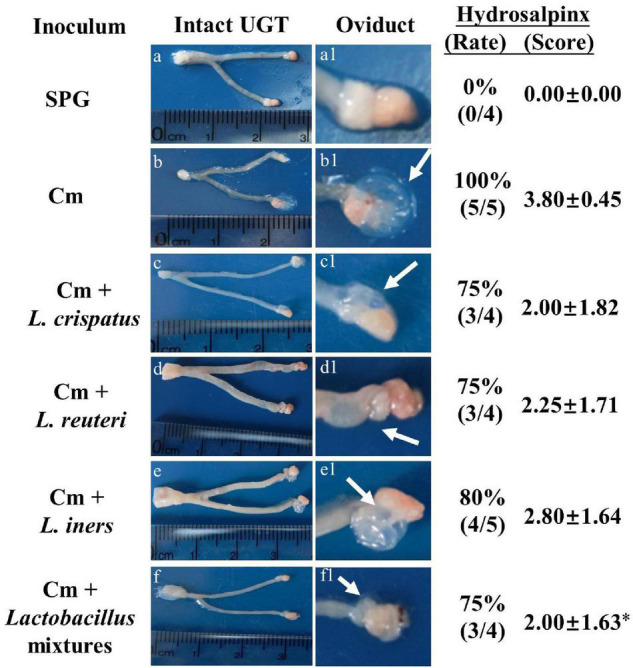
Detecting gross genital tract pathology from lactobacillus intravaginally administrated mice following intravaginal infection with *C. muridarum*. Groups of female BALB/c mice (*n* = 5) intravaginally inoculated with different lactobacilli as described in the Figure 3 legend. On Day 80 after infection, all the mice were sacrificed for genital tissue pathology evaluation. One representative overall appearance of the entire upper genital tract was displayed on the left column **(a-f)**, while the magnified images of the oviduct on the **(a1–f1)**, with the corresponding hydrosalpinx marked with white arrows. The corresponding hydrosalpinx incidence rate (Chi-square test) and severity scores were given on the right column. *p < 0.05 vs. the Cm group (one-way ANOVA).

**FIGURE 6:**
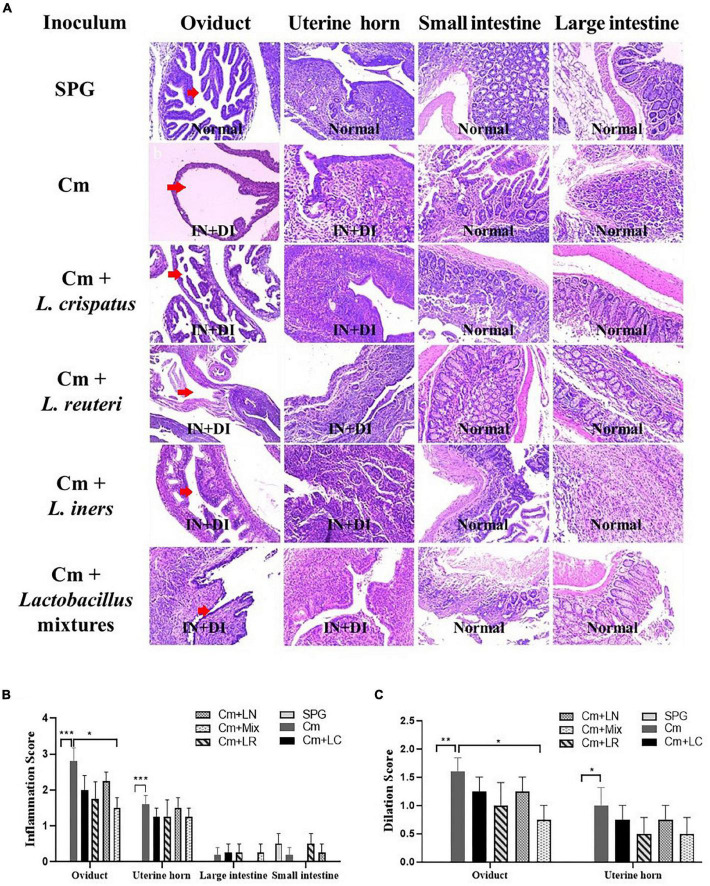
Effects of lactobacillus intravaginal administration on chlamydia-induced mouse genital and intestinal tract pathologies, following intravaginal infection with *C. muridarum*. **(A)** The groups of the mice were treated as described in the Figure 3 legend. The urogenital tract tissues were subjected to histological section analyses for observing inflammatory pathologies *via* H&E staining. Sections of oviduct and uterine horn tissues from the mice in each group were examined for both inflammatory infiltration (IN) and lumenal dilatation (DI) (red arrows), whereas small and large intestine tissues for inflammatory infiltration. The extent of inflammation **(B)** and dilatation **(C)** was semiquantitatively scored as described in the method section. The scores were calculated as the means and standard deviations for each group. *p < 0.05, **p < 0.01, and ***p < 0.001 vs. the Cm group (one-way ANOVA).

Eighty days after the infection, all the mice were sacrificed to evaluate the pathology in mouse genital tract tissues. We found that the mice intravaginally inoculated with *C. muridarum* alone developed typical pathological changes in their genital tracts, with no significant difference in the incidence of hydrosalpinx formation among lactobacillus-administrated groups (Figure 5). When the severity of hydrosalpinx was semi-quantitatively scored, a lower score in the lactobacillus mixture-inoculated mice was observed as compared to those intravaginally inoculated with *C. muridarum* alone. The gross upper genital tract pathology was further confirmed, following microscopic examination. Reduced levels of inflammatory infiltrate and luminal dilation were observed in the oviduct but not uterine horn tissue from the lactobacillus mixture-inoculated mice (Figure 6). Besides, the lactobacillus mixture-inoculated mice had comparable levels of live organism shedding, chlamydia-induced mouse genital inflammatory pathology, as well as *C. muridarum*-mediated Th1-types cytokines levels to those given lactobacillus mixture inoculation 3 days before chlamydia infection (Supplementary Figure 3), indicating which could be developed as a potential prophylactic agent for chlamydia. It was worth noting that we did not find any typical inflammatory cell infiltrate in the small and large intestines from the mice in each group (Figure 6C), indicating that chlamydial colonization in the intestinal tract was unable to induce intestinal inflammation, and single or mixed lactobacillus intravaginal inoculation had no impact in this situation.

### *Chlamydia muridarum*-Mediated Th1-Dominant Immunity Can Be Reduced by Lactobacillus Mixtures

**FIGURE 7:**
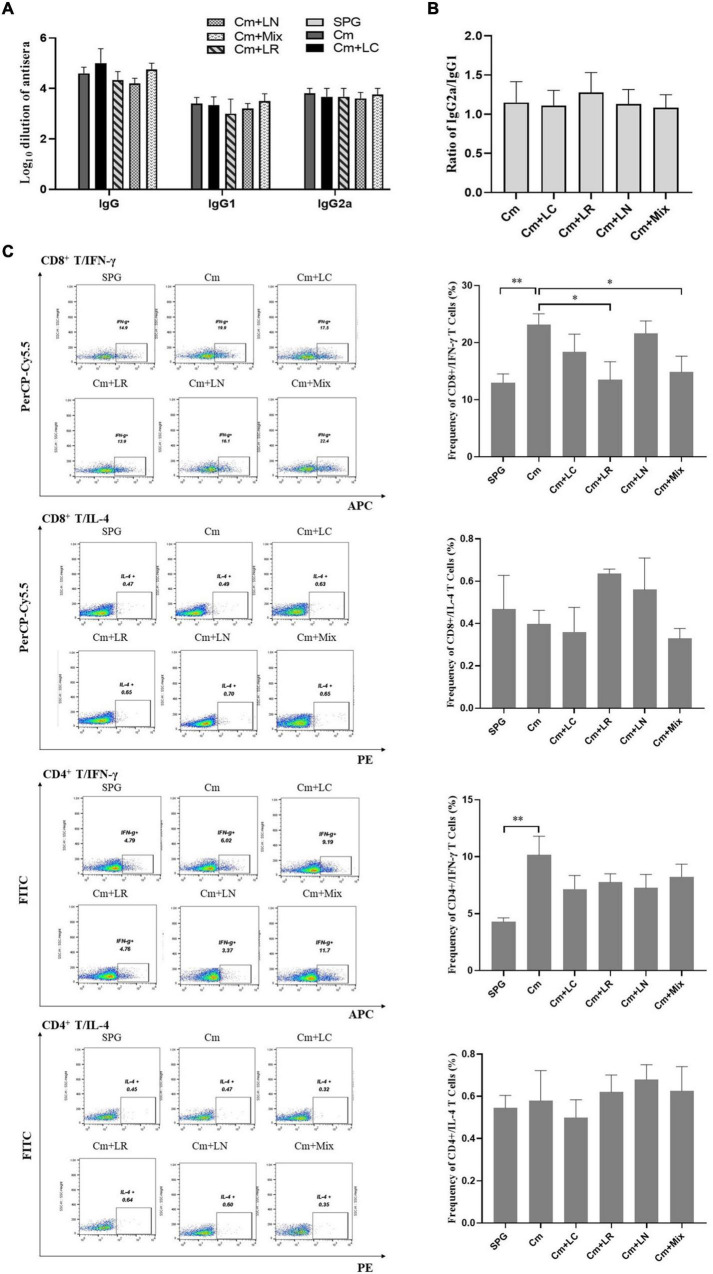
Effects of lactobacillus intravaginal administration on the switch of chlamydia-mediated immune responses, following intravaginal infection with *C. muridarum*. On Day 80 after infection, mouse sera from the six groups of the mice were collected to measure the titers of chlamydia-specific total IgG Abs and IgG in different isotypes **(A)**. A 10-fold serially dilutions of the mouse sera were carried out, starting with 1:10; the Ab titers of which were expressed as log10 dilution. **(B)** The ratio of IgG2a versus IgG1 from each group of mice was calculated (inapplicable for the SPG group). **(C)** Spleens were harvested from each mouse at Day 80, and 2- × −10^6^ splenocytes were cultured with a cell stimulation cocktail at 37°C for 6 h. Following stimulation, intracellular IL-4 and IFN-γ staining was carried out and determined by flow cytometry as detailed in section “Materials and Methods.” Frequency of CD8^+^/IFN-γ, CD8^+^/IL-4, CD4^+^/IFN-γ, and CD4^+^/IL-4 cells was calculated according to intracellular flow cytometry staining. Results from an independent sample. *p < 0.05 and ^**^p < 0.01 (one-way ANOVA).

To determine whether lactobacillus intravaginal inoculation has impact on *C. muridarum-*induced immunity, mice sera were analyzed by ELISA for chlamydia-specific total IgG antibody titers. All the mice, irrespective of the lactobacillus administration, developed robust antibody responses to *C. muridarum* compared to the control group, with a high ratio of IgG2a versus IgG1 (more than 1). There was no significant difference in the titers of chlamydia-specific antibody, including IgG, IgG1, IgG2a, and the ratio of IgG2a/IgG1 among each group (Figures 7A,B). We further monitored the phenotypic characteristics of T-cell populations using an *in vitro* spleen cell restimulation assay *via* intracellular flow cytometry staining analysis. Splenocytes were harvested from the mice infected with *C. muridarum* alone, secreting significant levels of CD8^+^/IFN-γ and CD4^+^/IFN-γ but not CD8^+^/IL-4 and CD4^+^/IL-4 upon restimulation with chlamydial antigens as compared to control groups. Both single *L. reuteri* and *L. iners*-inoculated mice secreted comparable CD8^+^/IFN-γ and CD4^+^/IFN-γ to the *C. muridarum* alone-infected mice, while the single *L. crispatus* and lactobacillus mixture-inoculated mice secreted a lower level of CD8^+^/IFN-γ (Figure 7C and Supplementary Figure 4). These data have demonstrated that lactobacillus intravaginal inoculation has neither affected mouse overall antibody response to *C. muridarum* infection nor altered the type of *C. muridarum*-mediated immune response, however, which attenuated *C. muridarum*-mediated Th1-dominant immunity.

### Alterations of Vaginal and Intestinal Microbiota Correlates With *Chlamydia muridarum*-Mediated Upper Genital Tract Pathology in Mice

**FIGURE 8:**
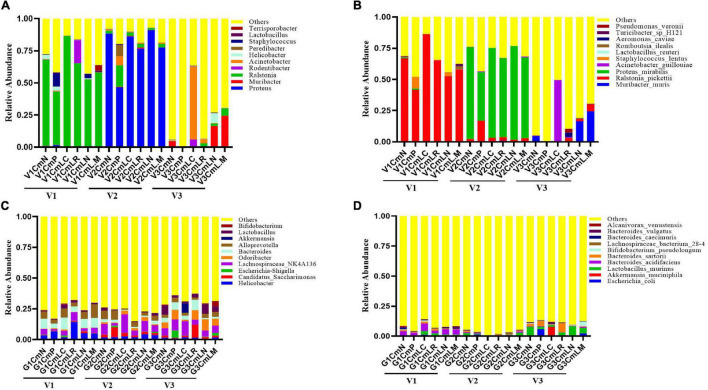
Taxonomic composition of the vaginal and intestinal microbiota. Both vaginal and intestinal swab samples were carefully collected at Visit 1 (V1: pre-medroxyprogesterone injection and chlamydia infection), Visit 2 (V2: 14 days after chlamydia infection), and Visit 3 (V3: 80 days after chlamydia infection). Stacked bar charts of top 10 taxa in terms of relative abundances at the genus **(A,C)** and species **(B,D)** levels for vaginal and intestinal microbiota, respectively. Remaining taxa are grouped in the “Other” category. Each mouse was intravaginally inoculated with SPG alone (CmN), or live *C. muridarum.* On Day 3 postinfection, the mice were given either an MRS medium (CmP), *L. crispatus* (CmLC), *L. reuteri* (CmLR), *L. iners* (CmLN), or a mixture of these bacteria (CmLM) at a ratio of 1:1:1 intravaginal inoculation.

The vaginal and intestinal tracts harbor multitudinous microorganisms that exist in a mutualistic relationship with the host, protecting the host against invading pathogens (Chee et al., 2020). To further demonstrate the effect of vaginal and intestinal microbiota shifts on *C. muridarum* infection, a total of 86 vaginal and 86 intestinal samples were collected at Visit 1 (V1: pre-medroxyprogesterone injection and chlamydia infection), Visit 2 (V2: 14 days after chlamydia infection), and Visit 3 (V3: 80 days after chlamydia infection) for 16S rRNA amplicon sequencing (4 vaginal and 2 intestinal samples were excluded because they had failed to pass the sequencing and quality control). The Good’s coverage index value of each sample exceeded 98% (Supplementary Tables 1, 2), as well as the rarefaction curve of each one, displayed an asymptote after a sharp rise (Supplementary Figures 5A, 6A), suggesting that the sampling and sequencing data were reasonable and sufficient. The vaginal microbiota of all the mice was mainly dominated by Ralstonia (41–86%) at Visit 1 and Proteus (46–91%) at Visit 2, with no significant difference observed in relation to genus distribution among each group (Figure 8A). PCoA based on Bray–Curtis dissimilarities showed no significant segregation of the groups within each visit; however, vaginal and intestinal samples at each visit were clustered and separated from the other visits (Supplementary Figures 5B, 6B). There is a significant shift on the vaginal microbiota of the SPG control mice observed between Visits 1 and 3. While the Muribacter was more abundant in lactobacillus mixture group (25%) than that in *C. muridarum*-alone group (.1%) at Visit 3 (Figure 8C). Similar results were also found at the species level. The corresponding *Muribacter muris* was significantly enriched in the lactobacillus mixture group (24%) compared with the *C. muridarum*-alone group (.06%) at Visit 3.

For intestinal microbiota, no significant difference was observed about genus distribution among each group at Visit 1 and Visit 2, while both the genus Bifidobacterium and corresponding *Bifidobacterium pseudolongum* were remarkably enriched in the lactobacillus mixture group compared with the *C. muridarum*-alone group at Visit 3 (Figures 8B,D). In addition, the heatmap representing log10-transformed relative abundances of microbial taxa highlights the variation of microbial composition among the individuals and the diversity in all vaginal and intestinal bacterial communities (Supplementary Figures 5C, 6C). These data indicated that shifts of *Muribacter muris* in the vaginal microorganism and *Bifidobacterium pseudolongum* in the intestinal microorganisms may be correlative with chlamydia clearance and chlamydia-mediated pathologies.

## Discussion

Chlamydia infection affects both men and women, occurring across all racial and ethnic groups, which is recognized as the most widely reported bacterial sexually transmitted infection (STI) and causes significant health and economic costs around the world (Golden et al., 2005). Women are likely to have more serious health problems from STIs than men (Kreisel et al., 2021), although their vagina harbors a large number of microorganisms, establishing vaginal micro-environment, which played a pivotal role in protecting against foreign pathogenic germs. By producing bacteriocin and species metabolites, and maintaining a low unfriendly pH (Fayol-Messaoudi et al., 2005; Miller et al., 2016), a healthy lactobacillus-dominated vaginal microbiota helps to maintain vaginal health and reduce the incidence of STIs.

Based on our recent findings that women with positive *C. trachomatis* have a change in their vaginal microbiota, with a marked decrease in lactobacillus dominated by *L. iners* (Chen et al., 2021), we now report that these altered lactobacilli are capable of preventing chlamydia infection both *in vitro* and *in vivo.* First, we employed lactobacillus species, including *L. iners, L. crispatus, L. jensenii, L. salivarius, L. gasseri, L. mucosae*, and *L. reuteri*, which showed a significant alteration in vaginal microbiota in women with *C. trachomatis* infection. The seven lactobacillus CFS inhibited *C. trachomatis* infectivity in dose- and time-dependent manners. Of note, each of these seven lactobacilli has different anti-chlamydia potency, ranging from 50 to 90%, with *L. crispatus* being the most potent. Second, over 40 components of lactobacillus CFS were examined, with lactic acid emerging as the most important product. Lactic acid isomers were discovered to have anti-chlamydia properties similar to lactobacillus CFS. Third, the mice intravaginally inoculated with lactobacillus mixtures or *L. crispatus* alone significantly reduced the level of live organism shedding course following genital chlamydia infection as compared to those who were not. The reduced numbers of recovered live organisms may facilitate the clearance of chlamydia in the lower genital tract, indicating that lactobacillus inoculation post infection may alter the course of infection in a positive manner, reducing EB shedding duration and inflammation. Fourth, we found that lactobacillus mixtures intravaginal inoculation significantly reduced chlamydia-induced cytokines production (TNF-α, IFN-γ, and IL-1β) in the vagina, as well as the severity of hydrosalpinx, oviduct inflammation, and dilatation. Fifth, the lactobacillus mixture-inoculated mice generated equal levels of chlamydia-specific antibodies to those infected with chlamydia alone. However, splenocytes from those mice produced lower levels of Th1 cytokines CD8+/IFN-γ and CD4+/IFN-γ, indicating that it only affected the intensity of *C. muridarum*-mediated Th1-dominant immunity, but not the type. Finally, 16S rRNA amplicon sequencing data demonstrated that vaginal *Muribacter muris* and intestinal *Bifidobacterium pseudolongum* may be linked to chlamydia-mediated pathologies in the upper genital tract.

Lactobacillus species are probiotics that protect women against invading pathogens and opportunistic illnesses as the first line of defense (Chee et al., 2020). During genital *C. trachomatis* infection, the relative abundance of vaginal lactobacillus changed dramatically, with the majority decreasing (*L. crispatus*) and others increasing (*L. iners*) (Chen et al., 2021). We chose the top seven lactobacillus strains with substantial changes for this investigation to assess their potential effects on *C. trachomatis* infection. *In vitro*, they both had a significant inhibitory effect on chlamydia infectivity. As a result, we examined the amounts of 45 metabolic components in lactobacillus CFS that could be linked to chlamydia inactivation. We found that D (−) lactic acid produced by lactobacillus was the most effective antibiotic to inactive *C. trachomatis* organisms, and positively correlated with the inhibitory activity. This finding is in accordance with previous studies (Nardini et al., 2016; Edwards et al., 2019).

Moreover, 2–100 mM D (−) lactic acid, covering the average concentration of that in the cervicovaginal fluid in women (O’Hanlon et al., 2013), was found to have either partially or fully anti-chlamydia activity, which was almost completely lost when their acidity was neutralized, indicating that it was not the protonation state only that determines the level of protection, and lactobacillus-produced endogenous D (−) lactic was effectively for inactivating *C. trachomatis* at a native pH. *L. crispatus* produces D (−) lactic acid efficiently in protecting against *C. trachomatis* infection in women, which was partially explained by the fact that vaginal microbiota dominated by *L. crispatus* significantly reduced the risk of chlamydial STI when compared to those dominated by *L. iners*, which barely produces D (−) lactic acid (van Houdt et al., 2018; Edwards et al., 2019).

Chlamydia lacks several metabolic enzymes; therefore, they must parasitize within the host cell to carry out their developmental cycle (Omsland et al., 2014; Rother et al., 2019). The HeLa cells were exposed to D (−) lactic acid to further examine their impacts on cell viability, and they showed similar cell vitality to those exposed to DMEM media, indicating that D (−) lactic acid exposure did not affect HeLa cell viability. Other studies, on the other hand, have found that lactobacillus-produced D (−) lactic acid was able to inhibit epithelial cell proliferation *via* downregulation of cyclin D1 and cyclin E1 production (Matsuki et al., 2013; Edwards et al., 2019). This inhibition may prevent chlamydia from entering mucosal epithelial cells and surviving there. Recently, studies have also provided evidence that D (−) lactic acid produced by certain lactobacillus had *in vitro* inhibitory activity against bacterial, protozoan, and viral STIs, including Group B Streptococcus, *Listeria monocytogenes*, and HIV (Gravesen et al., 2004; Fayol-Messaoudi et al., 2005; Tyssen et al., 2018). In this context, we postulated that direct exposure of chlamydia EBs or host cells to D (−) lactic acid could protect against chlamydia infection by disturbing the integrity of the chlamydial outer membrane and lowering the adhesion of chlamydia EBs to the host cell, respectively. An effort is underway to uncover the mechanisms by which D (−) lactic acid inactivates *C. trachomatis* organism. Besides, *L. crispatus* biosurfactants were capable of reducing 50% of chlamydia EB infectivity toward the HeLa cells, which indicated the promising effects of lactobacilli-derived biosurfactants on anti-chlamydial strategies (Foschi et al., 2021).

We noticed that both *L. crispatus* and *L. reuteri* had substantial anti-chlamydial activity *in vitro*. Although *L. iners* exhibited the lowest inhibitory properties, it dominated the vaginal microbiota, following chlamydia infection in women (Filardo et al., 2017). Additionally, lactobacillus inoculation 3 days after postinfection in the female BALB/c mice was reported to inhibit the growth of *G. vaginalis* (Daniele et al., 2014). Thus, we sought to deepen the knowledge about their anti-chlamydial properties *in vivo*. For this purpose, the mice were intravaginally inoculated with the three single lactobacilli or their mixtures 3 days after genital chlamydia infection. The single lactobacillus-inoculated mice, with the exception of *L. crispatus*, developed a similar level of live chlamydia organisms shedding from a lower genital tract to those without. While the lactobacillus mixtures-inoculated mice had a lower level, indicating a lower infectious titer of chlamydia in the mouse lower genital tract. This reduced chlamydia shedding, which suggested that there are less bacteria leading to attenuated inflammation and a Th1 immune response, which implied that colonization with lactobacillus could be impacting the immune response. We, thus, postulated that mixed lactobacilli could play a synergistic role in anti-chlamydia. It will be worthwhile to put forth the effort to test the aforementioned possibilities.

Colonization of chlamydia in the gastrointestinal tract has been linked to increased chlamydial pathogenicity in the upper genital tract (Tian et al., 2020; Zhong, 2021). Hence, we monitored the live chlamydial organism shedding in the gastrointestinal tract and found that intravaginally inoculated chlamydia could be detected in the mouse rectal swabs within as early as 3 days, suggesting rapid spread of chlamydia from the lower genital tract to the gastrointestinal tract. Moreover, chlamydia could have established long-term colonization and achieved a certain level of fitness with the gastrointestinal tract. It was surprising to us because this colonization did not cause any pathologies in the large or small intestine, which may serve as a reservoir for chlamydia (Lee et al., 2015; He et al., 2021). We found that lactobacillus mixtures inoculation reduced the spread of genital *C. muridarum* to the gastrointestinal tract, which may be due to the low quantity of infectious progeny in the corresponding lower genital tract. As a result of the lower colonization in the gastrointestinal tract, which could, in turn, attenuate chlamydial pathogenicity in the upper genital tract (Tian et al., 2021), the way that chlamydia spreads from the vaginal tract to the gastrointestinal tract has attracted people’s interest. It may go through tissue penetration, lymph circulation, or a spleen-to-stomach pathway for hematogenous chlamydia to reach the large intestine lumen, whereas the precise mechanisms remain unknown (Zhong, 2018; Zhou et al., 2021).

In addition, *C. trachomatis* infections have also been reported to cause an alteration of vaginal microbiota (Filardo et al., 2017; Chen et al., 2021). To identify the possible microorganism candidates associated with chlamydia infection in mice, we employed a *C. muridarum*-infected mouse model—a useful model of genital chlamydia infections (Li et al., 2012), and profiled the relative abundance of microorganism in both mice vagina and gut. There is a significant alteration of *Muribacter muris* in the vagina and *Bifidobacterium pseudolongum* in the gut among lactobacillus mixture-administrated mice, which might affect chlamydia susceptibility; *Muribacter muris*, on the other hand, is an acid producer, which could exhibit increased tolerance to acidity produced by the intravaginally inoculated lactobacillus (Nicklas et al., 2015). In this study, vaginal microbiota of the mice that had no chlamydia or lactobacillus administration had also changed so much over time (visits 1–3). Huang et al. (2019) reported vaginal microbiota can vary on short-time scales, while gut microbiota does not change radically over short time scales generally. Low sample numbers and time-point effects may explain the change of vaginal microbiota.

It is worth noting that the composition of vaginal microbiota in human differs from that in mice in that the former often has > 70% resident lactobacillus, while the latter contains only < 1% (Miller et al., 2016). Lactobacillus may thrive in a favorable environment created by human physiology and high glycogen levels in the vaginal canal (Amabebe and Anumba, 2018). This could partially explain why we observed abundant lactobacillus on Day 4 and nearly undetectable lactobacillus on Day 10 after lactobacillus intravaginal inoculation in the present study. Although lactobacillus-inoculated mice did not develop long-lasting colonization of lactobacillus in the lower genital tract, it still exerted anti-Chlamydia properties *in vivo* experiments. This finding was further reinforced by the observation that relatively brief exposure of chlamydia EBs to lactobacillus CFS reduced *C. trachomatis* infectivity.

In summary, the findings reported here suggest that different lactobacillus strains exhibit varying anti-chlamydia potencies mainly by producing D (−) lactic acid, with *L. crispatus* being the most potent. *In vivo*, lactobacillus intravaginal inoculation protects against chlamydia infection by attenuating live chlamydia organisms shedding, cytokines production, and Th1-dominant immunity, ultimately reducing pathogenicity in the upper genital tract. Lactobacilli have exerted anti-chlamydia activity. Nevertheless, the majority of lactobacillus species were sensitive doxycycline and azithromycin, which were recommended regimen for chlamydial infection. Precise antibiotic treatment, including dose and type of antibiotic, should be taken into account to protect and restore optimal lactobacillus, following susceptibility test (Tamarelle et al., 2020; Workowski et al., 2021). This study shed light on the development of lactobacillus intravaginal administration as a therapeutic strategy for genital chlamydia infection.

## Data Availability Statement

The datasets presented in this study can be found in online repositories. The names of the repository/repositories and accession number(s) can be found below: NCBI BioProject – PRJNA808632.

## Ethics Statement

The animal study was reviewed and approved by the Ethics Committee of Chenzhou No. 1 People’s Hospital.

## Author Contributions

HC and ZL: conceptualization and funding acquisition. SM, LW, QZ, FL, HC, and LZ: methodology. HC, SM, and LZ: software. HC, LW, QZ, and SM: *C. trachomatis* infectivity assays. SM, LL, YW, WL, FL, and LP: mouse administration. HC and SM: writing—original draft preparation. ZL: writing—review and editing. All authors: contributed to the article and approved the submitted version.

## Conflict of Interest

The authors declare that the research was conducted in the absence of any commercial or financial relationships that could be construed as a potential conflict of interest.

## Publisher’s Note

All claims expressed in this article are solely those of the authors and do not necessarily represent those of their affiliated organizations, or those of the publisher, the editors and the reviewers. Any product that may be evaluated in this article, or claim that may be made by its manufacturer, is not guaranteed or endorsed by the publisher.
