# Supplementary material for: Lactobacillus Modulates Chlamydia Infectivity and Genital Tract Pathology in vitro and in vivo
Source: Front Microbiol. 2022 Apr 28;13:877223. doi: 10.3389/fmicb.2022.877223 (PMC9098263; doi:10.3389/fmicb.2022.877223)
Supplement: Supplementary file 1 [file Data_Sheet_1.PDF]

Lactobacillus modulates Chlamydial infectivity and genital tract pathology in female mice

Supplementary Tables and Figures

Table S1. Alpha diversity index for each vaginal swab samples

	Observed species	shannon	simpson	chao1	ace	Goods coverage
V1CmN1	268	0.735863	0.166104	431.1974	525.8098	0.990953
V1CmN2	170	0.86252	0.24011	277.3125	367.2541	0.994159
V1CmN3	621	1.168584	0.224119	1085.83	1217.282	0.976809
V1CmN4	1645	3.265528	0.524068	1946.687	2073.481	0.962838
V1CmN5	199	5.052118	0.944337	233.45	264.8307	0.996965
V1CmP1	1022	3.389298	0.500597	1344.738	1490.449	0.976695
V1CmP3	342	5.269472	0.9279	433.2941	442.4588	0.994446
V1CmP4	638	5.70141	0.945035	753.4698	822.4934	0.98935
V1CmP5	83	0.460456	0.095317	140.4	146.0398	0.997595
V1CmLC1	275	1.109414	0.256094	412.0143	515.0574	0.992041
V1CmLC2	177	0.550914	0.129006	290.2941	342.2979	0.993816
V1CmLC3	341	1.02671	0.248896	437.5714	513.114	0.990323
V1CmLC4	1408	2.055332	0.321012	2807.903	3255.184	0.943369
V1CmLC5	522	1.448539	0.296056	825.2517	1012.153	0.983108
V1CmLR1	230	0.732296	0.178688	377.7031	474.9998	0.992098
V1CmLR2	253	1.249354	0.247303	360.0536	414.0805	0.993701
V1CmLR3	180	1.012024	0.205556	255.1463	294.0673	0.995476
V1CmLR4	1423	2.775816	0.419053	1626.447	1726.943	0.971026
V1CmLR5	612	2.643969	0.470623	739.4603	859.0638	0.987403
V1CmLN1	625	1.342595	0.268762	1273.007	1709.219	0.975149
V1CmLN2	722	1.396809	0.261541	1194.548	1335.509	0.974462
V1CmLN3	1684	8.932615	0.993612	1952.932	2050.989	0.975092
V1CmLN4	506	4.881476	0.928365	700.487	808.8836	0.987861
V1CmLN5	15	0.430426	0.144703	17	20.17943	0.999771
V1CmLM1	579	1.54437	0.31399	697.7132	830.7221	0.985799
V1CmLM2	1645	3.386179	0.527902	1887.227	2040.664	0.965873
V1CmLM3	900	5.170168	0.914234	1238.638	1421.921	0.977668
V1CmLM4	2047	4.722289	0.692557	2062.799	2138.275	0.987975
V1CmLM5	424	1.104745	0.255957	877.3789	1042.064	0.983165
V2CmN1	53	0.89185	0.312662	89.11111	106.4266	0.998511

V2CmN2	186	1.728607	0.43621	236.4	263.9628	0.996335
V2CmN3	223	2.248242	0.57029	310.2083	350.1998	0.994732
V2CmN4	99	1.205778	0.361997	150.75	173.6842	0.997366
V2CmN5	235	1.925891	0.471545	355	364.7549	0.994503
V2CmP1	213	2.830935	0.660479	260.7818	294.1989	0.99582
V2CmP2	195	2.84315	0.640617	230	257.5261	0.996793
V2CmP3	112	3.617475	0.834553	167.7143	173.9955	0.99771
V2CmP5	158	1.580485	0.417794	211.0526	260.4421	0.996335
V2CmLC1	214	2.094436	0.51229	276.5625	309.4497	0.995534
V2CmLC2	84	1.857211	0.515164	109.5882	125.0969	0.998282
V2CmLC3	67	1.144667	0.361536	96.54545	100.6589	0.998511
V2CmLC4	181	1.507089	0.397431	221.2273	241.2537	0.996564
V2CmLC5	195	1.892122	0.497627	266.1429	312.4481	0.99519
V2CmLR1	111	1.49822	0.429845	144.1154	167.7439	0.997595
V2CmLR2	314	4.072498	0.804631	364.4483	382.328	0.995591
V2CmLR3	201	2.217549	0.53162	245.8	270.4807	0.996335
V2CmLR4	215	2.360769	0.553898	264.9149	295.9212	0.996049
V2CmLR5	159	1.954207	0.488325	181.4545	196.3129	0.997767
V2CmLN1	166	1.470835	0.382509	204.0769	233.194	0.996851
V2CmLN2	144	1.50616	0.421761	201.0333	218.6757	0.996622
V2CmLN3	240	1.882117	0.464306	306.4545	337.5962	0.995076
V2CmLN4	131	1.371466	0.393445	167.12	176.4675	0.997538
V2CmLN5	95	1.315529	0.392699	132	133.1907	0.997881
V2CmLM1	314	3.046253	0.605085	354.4717	358.8886	0.996221
V2CmLM2	337	3.445301	0.664842	455.6957	453.2938	0.993988
V2CmLM3	164	1.597889	0.40765	210.4063	223.0413	0.996851
V2CmLM4	301	2.201432	0.493012	372.6308	403.355	0.994446
V2CmLM5	269	2.869452	0.615834	309.4483	334.156	0.996049
V3CmN1	1916	9.313866	0.995877	2305.107	2379.866	0.969537
V3CmN2	2067	9.363517	0.995315	2499.374	2599.388	0.965586
V3CmN3	2019	9.407708	0.996094	2417.496	2508.013	0.967762
V3CmN4	923	4.943777	0.888581	1293.222	1421.417	0.977554
V3CmN5	2063	9.412466	0.996174	2557.411	2677.25	0.963468
V3CmP1	2046	9.467917	0.996283	2339.311	2446.335	0.970454
V3CmP2	2002	9.359628	0.995948	2323.142	2481.183	0.968678
V3CmP3	1986	9.391169	0.996107	2280.631	2414.106	0.970683
V3CmP4	2108	9.466183	0.996413	2622.122	2717.814	0.962838
V3CmLC1	2561	9.754873	0.996948	3268.16	3515.13	0.948694
V3CmLC2	335	1.927867	0.453614	451.3187	513.7734	0.99164
V3CmLC3	369	1.944152	0.435443	501.2211	545.7149	0.990896
V3CmLR1	1946	9.371496	0.996017	2206.777	2321.299	0.972572
V3CmLR2	1844	8.102075	0.974651	2158.847	2300.757	0.969423

V3CmLR3	2179	9.421627	0.995199	2675.369	2820.195	0.961235
V3CmLN1	2021	9.375932	0.99577	2345.858	2491.48	0.968736
V3CmLN2	597	4.88017	0.878822	724.4444	787.2675	0.98935
V3CmLN3	1978	9.326207	0.995336	2295.165	2422.445	0.969824
V3CmLN4	1993	9.411244	0.995784	2226.513	2348.041	0.97303
V3CmLN5	217	1.771784	0.36736	277.3261	305.858	0.995705
V3CmLM1	1955	9.135621	0.992138	2344.353	2457.775	0.967934
V3CmLM2	142	0.28619	0.04292	211.2564	238.3894	0.995763
V3CmLM4	1787	8.744059	0.98094	2008.142	2083.843	0.976466
V3CmLM5	2088	9.459137	0.995776	2443.385	2545.152	0.967877

Table S2. Alpha diversity index for each intestinal swab samples

	Observed species	shannon	simpson	chao1	ace	Goods coverage
G1CmN1	604	6.441816	0.97229	645.5714	671.2633	0.996631
G1CmN2	534	5.649867	0.95134	649.2805	660.6169	0.995207
G1CmN3	548	5.622592	0.948095	634.0424	692.443	0.995033
G1CmN4	483	6.128662	0.961832	517.7561	535.3762	0.99736
G1CmN5	368	5.675592	0.957297	413.1429	442.4008	0.997221
G1CmP1	521	5.349542	0.940352	659.1098	672.5193	0.994755
G1CmP2	483	5.768796	0.956667	533.5638	561.9592	0.996596
G1CmP3	468	4.968129	0.907718	567.1064	620.4083	0.995241
G1CmP4	538	5.632397	0.941073	646.5	719.6661	0.994616
G1CmP5	499	5.438569	0.943648	588.0805	607.131	0.995658
G1CmLC1	549	6.29866	0.972528	627.4706	649.1549	0.995971
G1CmLC2	499	5.294002	0.9204	587.2245	633.0144	0.995415
G1CmLC3	386	4.925877	0.927421	459.5814	511.516	0.996075
G1CmLC4	479	5.622545	0.949654	545.6105	583.1756	0.996075
G1CmLC5	492	5.4509	0.947107	566.0345	587.3829	0.99604
G1CmLR1	570	6.349209	0.973728	647.5581	666.3448	0.995971
G1CmLR2	577	6.15884	0.968847	671.9604	717.1547	0.995172
G1CmLR3	523	5.663351	0.917461	594.3333	614.731	0.996249
G1CmLR4	648	5.686042	0.942556	753.0076	801.7357	0.994199
G1CmLR5	615	5.504367	0.878442	692.6814	735.2389	0.99538
G1CmLN1	738	7.020228	0.978991	838.7863	886.9265	0.994338
G1CmLN2	730	6.065721	0.960148	897.5809	945.8502	0.992567
G1CmLN3	659	5.712033	0.950612	823.6667	881.5982	0.992741
G1CmLN4	644	5.583012	0.939669	743.5496	782.446	0.994373
G1CmLN5	554	5.796141	0.963207	723.9286	772.3371	0.993644
G1CmLM1	592	6.627381	0.976584	741.039	753.4451	0.99472

G1CmLM2	426	5.564083	0.939181	458.0488	481.6064	0.997464
G1CmLM3	530	5.587972	0.934233	649.6582	665.4967	0.995207
G1CmLM4	474	5.707002	0.949401	526.6974	542.4039	0.996874
G1CmLM5	561	6.205967	0.962696	628.9765	649.3901	0.996249
G2CmN1	872	5.654376	0.941783	1169.194	1313.813	0.988433
G2CmN2	729	6.628337	0.976409	958.125	1011.405	0.991837
G2CmN3	737	5.714371	0.948715	970.2658	1089.188	0.990552
G2CmN4	756	6.107579	0.966058	1014.675	1139.455	0.990274
G2CmN5	845	6.553528	0.971269	1088.69	1150.376	0.990344
G2CmP1	508	5.273487	0.916906	619.913	665.1737	0.994998
G2CmP2	421	5.167587	0.918748	516.8788	529.5567	0.996075
G2CmP3	577	5.561691	0.943836	724.4094	812.3109	0.993262
G2CmP4	368	5.274324	0.928682	458.0159	499.5789	0.996283
G2CmP5	487	5.449482	0.938147	620.2788	712.5748	0.994199
G2CmLC1	847	6.734438	0.974688	952.5048	1050.399	0.992706
G2CmLC2	859	5.874144	0.945602	1132.97	1239.909	0.989476
G2CmLC3	592	5.806613	0.952398	695.4146	742.6205	0.994443
G2CmLC4	869	5.892052	0.934423	1190.6	1309.479	0.988329
G2CmLC5	786	5.86242	0.959397	979.9441	1075.482	0.99083
G2CmLR1	739	6.596135	0.97058	915.2895	1020.162	0.991942
G2CmLR2	800	6.293132	0.963443	946.768	1032.592	0.991976
G2CmLR3	712	5.624039	0.950739	991.042	1097.123	0.99017
G2CmLR4	587	6.959546	0.981248	669.6	694.7762	0.995867
G2CmLR5	603	7.031114	0.986758	752.4316	812.3786	0.99413
G2CmLN1	664	6.85997	0.97677	804.5545	853.8144	0.99413
G2CmLN2	783	6.016036	0.947352	880.5319	950.0007	0.993331
G2CmLN3	729	6.690604	0.979087	906.1	1012.534	0.991976
G2CmLN4	1043	6.957545	0.977839	1424.005	1560.67	0.986697
G2CmLN5	569	6.480661	0.972539	639.6111	688.8359	0.995693
G2CmLM1	844	6.189485	0.962793	1006.657	1105.637	0.990969
G2CmLM2	1082	6.315141	0.963796	1447.755	1529.664	0.986176
G2CmLM3	893	6.522567	0.971665	1146.131	1229.899	0.989614
G2CmLM4	851	6.284908	0.969216	1039.723	1142.635	0.990656
G2CmLM5	913	6.295479	0.961637	1276.056	1425.451	0.98753
G3CmN1	551	5.51927	0.954027	636.3629	681.5931	0.994929
G3CmN2	526	6.494479	0.971362	573.6163	593.5881	0.996839
G3CmN3	564	5.595066	0.951366	699.7742	782.4943	0.993609
G3CmN4	742	5.764119	0.93655	913.75	990.3747	0.992046
G3CmN5	597	6.068351	0.969852	703.1628	761.6429	0.994234
G3CmP1	634	6.003701	0.957481	733.2707	793.1692	0.994338
G3CmP2	413	4.810672	0.884686	479.4474	501.9725	0.996492
G3CmP3	795	6.702375	0.979737	961.2069	1051.366	0.991629

G3CmP4	630	5.627449	0.925179	767.6281	816.7412	0.993644
G3CmP5	704	6.591143	0.976515	836.7083	917.9264	0.993192
G3CmLC1	677	6.593342	0.977485	807.5396	883.0441	0.993366
G3CmLC2	646	5.677854	0.941548	821.2903	869.98	0.992741
G3CmLC3	445	5.813866	0.96234	513.2432	536.0936	0.996492
G3CmLR1	486	6.271272	0.965404	533.7561	561.2691	0.996909
G3CmLR2	447	4.886677	0.886873	550.25	571.2997	0.995867
G3CmLR3	584	6.068015	0.96957	696.6911	766.3915	0.994199
G3CmLN1	342	5.280758	0.948693	403.6154	431.254	0.996874
G3CmLN2	417	5.674602	0.95908	461.0632	503.2008	0.996804
G3CmLN4	350	5.214113	0.941569	433.6379	453.0651	0.996561
G3CmLN5	532	5.498094	0.926212	573.1346	612.3931	0.99677
G3CmLM1	332	5.277215	0.952212	386.4688	413.7874	0.997082
G3CmLM2	357	5.221392	0.946891	401	440.397	0.996943
G3CmLM4	466	5.929187	0.954466	523.5132	545.5461	0.996735
G3CmLM5	306	5.551878	0.955521	356.0769	364.6324	0.997812

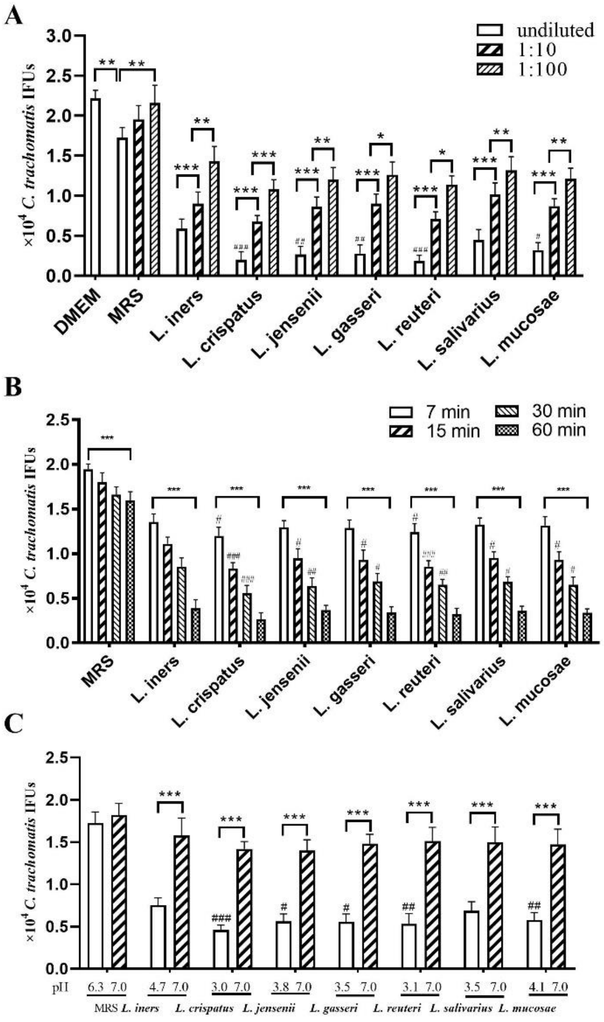

Figure S1. Concentration-, time- and pH- dependent inhibition of *C. trachomatis* infectivity by Lactobacillus culture supernatants. Lactobacillus supernatants were collected from overnight cultures of *L. iners, L. crispatus, L. jensenii, L. gasseri, L. reuteri, L. salivarius and L. mucosa. C. trachomatis* EB were pre-incubated with different dilutions (1:1, 1:10 and 1:100 diluted with PBS) of Lactobacillus culture supernatants (A), different time points: 7 minutes, 15 minutes, 30 minutes and 60 minutes (B) and different pH conditions: original pH of Lactobacillus supernatants and pH 7.0 (C). Following treatments, *C. trachomatis* IFUs were enumerated using a fluorescence microscopy. Each bar represents the mean ± SD of the IFUs from three independent experiments. *$P < 0.05$, ***$P < 0.01$ and ***$P < 0.001$; $^{\#}P < 0.05$, $^{\#\#}P < 0.01$ and $^{\#\#\#}P < 0.001$ vs corresponding *L. iners* culture supernatants treatment.

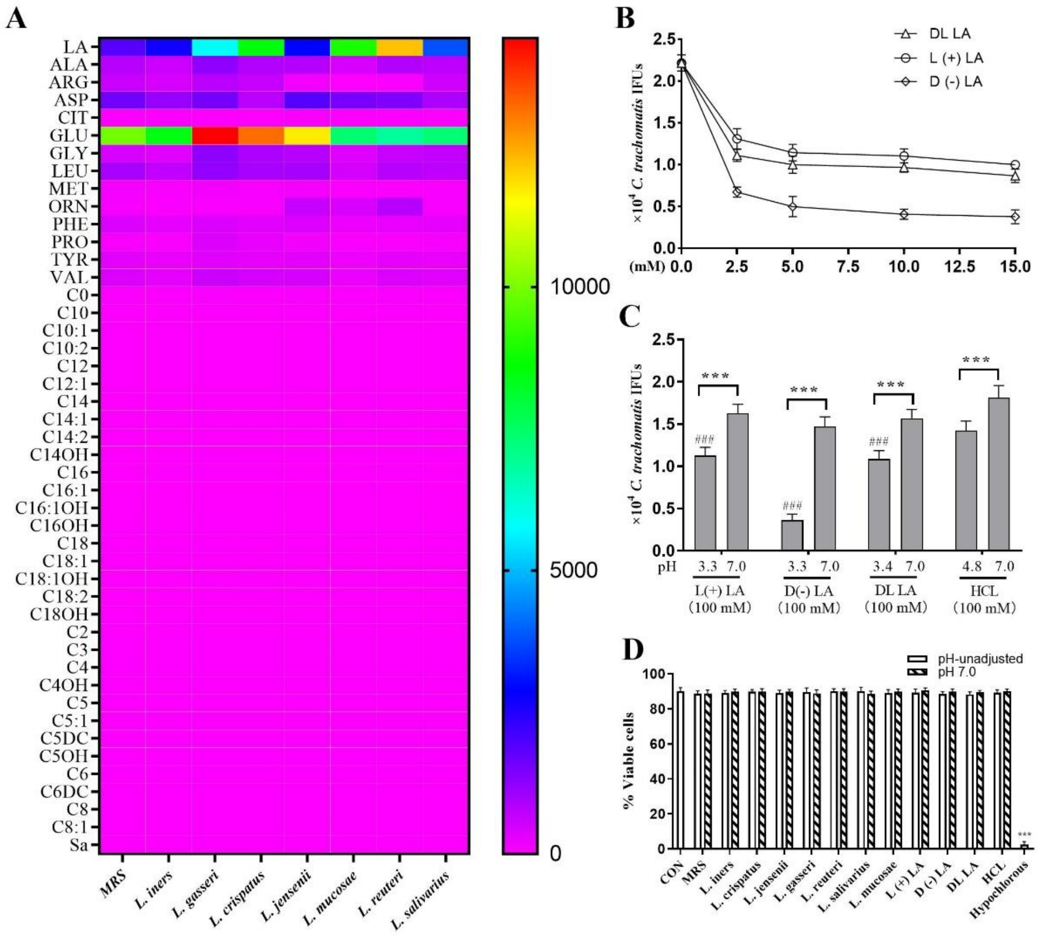

Figure S2. Effect of lactic acid isomers on *C. trachomatis* infectivity. (A) Detection of Lactobacillus metabolic components in Lactobacillus culture supernatants. Forty-five Lactobacillus metabolic components including lactic acid, non-derivatization of various amino acids and carnitine were assessed using ion selective electrode and UPLC-MS/MS. D (-), L (+) and DL lactic acid inhibited *C. trachomatis* infectivity in a dose- (B) and pH- (C) dependent manner. (D) Effects of Lactobacillus metabolites and lactic acid isomer on HeLa cells viability. Lactobacillus metabolites and lactic acid isomer were added to HeLa cells culture incubated for 24 h. After dying with trypan blue, HeLa cells viability were calculated. ***$P < 0.001$; $^{###}P < 0.001$ vs HCL.

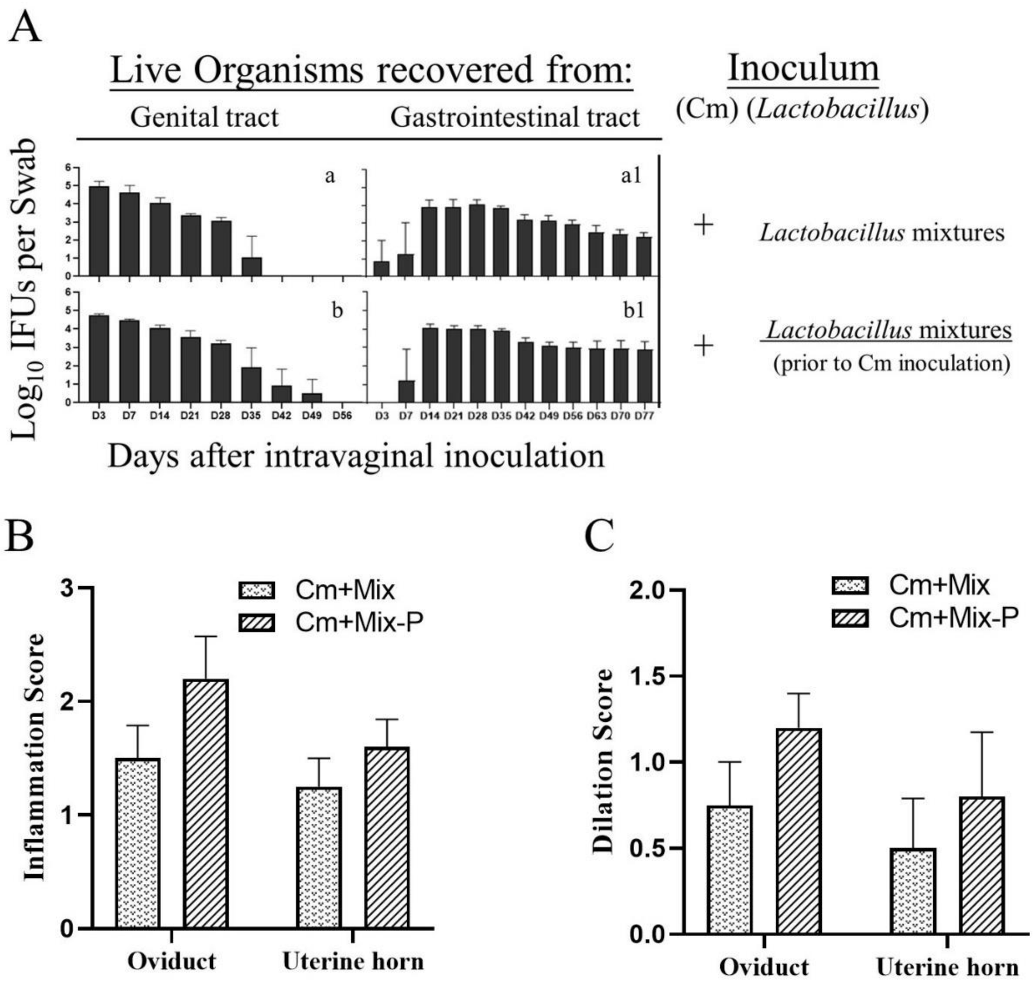

Figure S3. Effect of Lactobacillus mixture inoculation on the live organism shedding and Chlamydia-induced mouse genital inflammatory pathology. Mice were given Lactobacillus mixture inoculation 3 days before and after Chlamydia infection. (A) Comparison of on live organism shedding following chlamydial infection. The extent of inflammation (B) and lumen dilatation (C) of both uterine horns and oviducts was semiquantitatively scored under microscope.

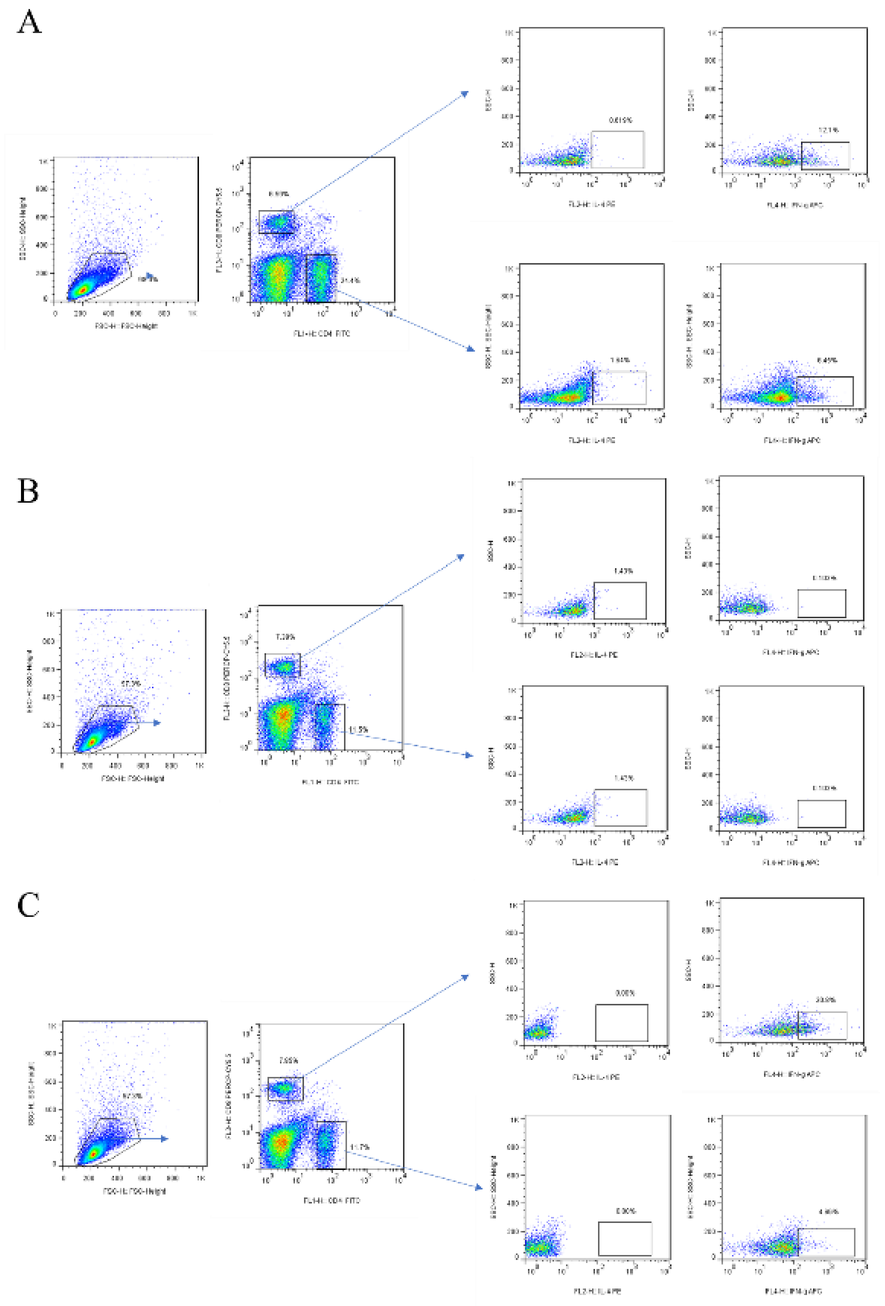

Figure S4. Gating strategy used to define the cytokines from T cells (A) and representative fluorescence-minus-one (FMO) control of IFN-γ (B) and IL-4 (C). Gating strategy for flow cytometry experiments assessing IFN-γ and IL-1β. $CD4^+$ and $CD8^+$ T cells were gated by SSC-A vs FSC-A, as indicated, and then the APC^+ and PE^+ population was plotted vs. SSC-A.

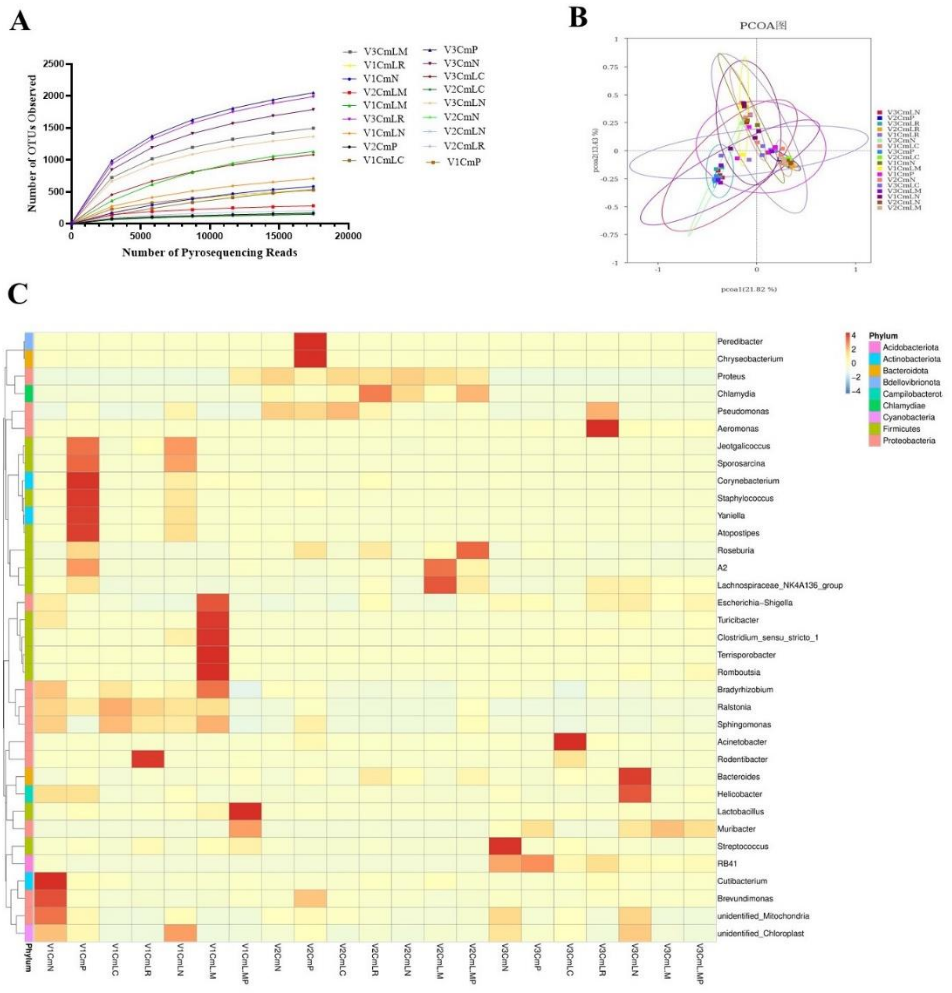

Figure S5. Vaginal microbial diversity. (A) Rarefaction curve of vaginal samples in each group, which tended to be gentle, indicating that the sequencing data was gradual and reasonable. (B) PCoA analysis based on weighted UniFrac distance. (C) Heatmap of beta-diversity index for each group, the legend below the heatmap represents each participant. Each mouse was intravaginal inoculated with SPG alone (CmN), or live *C. muridarum*. On day 3 postinfection, mice were given either MRS medium (CmP), *L. crispatus* (CmLC), *L. reuteri* (CmLR), *L. iners* (CmLN) or a mixture of these bacteria (CmLM) at a ratio of 1:1:1 intravaginally inoculation.

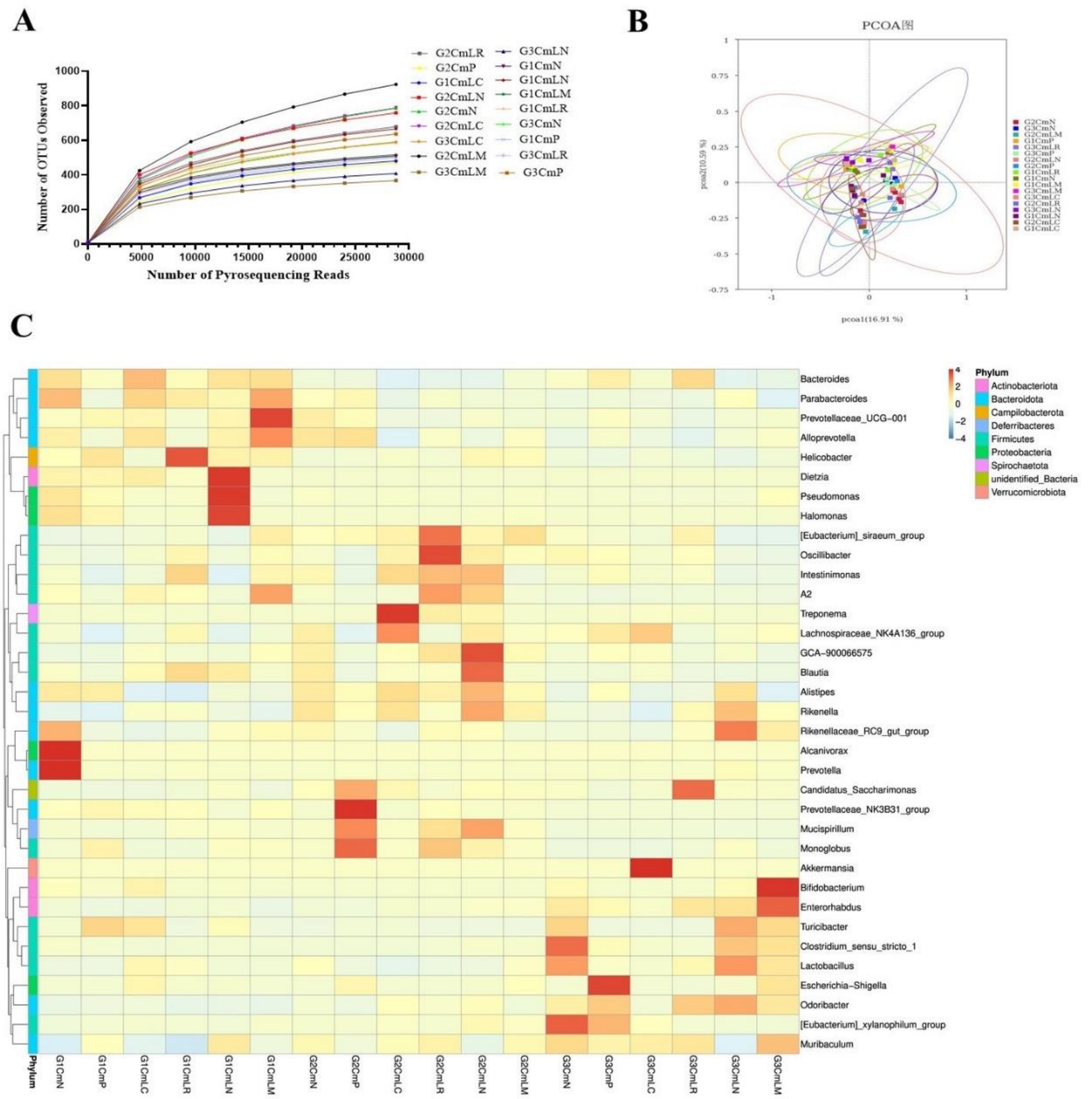

Figure S6. Intestinal microbial diversity. (A) Rarefaction curve of intestinal samples in each group, which tended to be gentle, indicating that the sequencing data was gradual and reasonable. (B) PCoA analysis based on weighted UniFrac distance. (C) Heatmap of beta-diversity index for each group, the legend below the heatmap represents each participant.
